# Co-occurrence of peritoneal mesothelioma and genitourinary cancers: a case series with comparative outcomes

**DOI:** 10.1515/pp-2025-0020

**Published:** 2025-11-03

**Authors:** Emma M. Bradley, James L. Rogers, Marissa C. Kuo, Deepa Magge

**Affiliations:** Division of Surgical Oncology and Endocrine Surgery, Section of Surgical Sciences, 12328 Vanderbilt University Medical Center, Nashville, TN, USA; Veterans Affairs Hospital, Tennessee Valley Healthcare System, Nashville Campus, Vanderbilt University Medical Center, Nashville, TN, USA; Vanderbilt University School of Medicine, Vanderbilt University Medical Center, Nashville, TN, USA

**Keywords:** peritoneal mesothelioma, CRS-HIPEC, genitourinary malignancy, multiple primary cancers, synchronous cancer, metachronous cancer

## Abstract

**Objectives:**

Peritoneal mesothelioma (PM) shares features with genitourinary (GU) malignancies, including histologic appearance, embryologic origin and genetic predispositions. However, data on their co-occurrence are limited. The study presents a case series of PM patients with associated GU malignancies and explores outcomes following cytoreductive surgery and hyperthermic intraperitoneal chemotherapy (CRS-HIPEC).

**Methods:**

A prospectively maintained CRS-HIPEC database from a tertiary referral center (2011–2024) was reviewed. Demographics, tumor characteristics and outcomes were compared between PM patients with and without GU malignancies (including gynecologic and urologic cancers).

**Results:**

Among 237 CRS-HIPEC patients, 8/17 patients with PM were found to have another GU malignancy (median age 52.8, 62.5 % male). This included renal cell carcinoma, prostate cancer, ovarian tumors and cervical carcinoma. Most GU malignancies were diagnosed before PM (5/8), two were diagnosed post-CRS-HIPEC, and one synchronously. Three patients reported asbestos exposure; two had *BAP1* mutations. Compared to those without GU malignancies, affected patients tended to have higher PCI (19.8 vs. 14.3) and poorer 3-year survival (62.5 vs. 100 %).

**Conclusions:**

GU malignancy is common among PM patients undergoing CRS-HIPEC and could represent a higher-risk subgroup. These findings raise the hypothesis of a potential association between PM and GU malignancy. Shared origins, oncogenesis of similar cell types, environmental exposures or genetic predispositions may contribute and warrant further investigation.

## Introduction

Mesothelioma is a rare and aggressive neoplasm originating from mesothelial cells that line the serosal surfaces of internal organs and body cavities [1]. Although the pleura is the most commonly affected site, mesothelioma may also involve the peritoneum, pericardium, or tunica vaginalis testis, albeit less frequently [2], [3], [4]. Cases of malignant mesothelioma affecting the uterus, ovaries and female genital tract have also been reported [5], [6], [7], [8]. The epidemiology and clinicopathologic behavior of extrapleural malignant mesotheliomas, particularly peritoneal mesothelioma (PM), are less well-defined compared to those of pleural mesothelioma.

First described in 1908, approximately 800 new cases of malignant PM are diagnosed annually in the United States [9]. While prognosis has been historically poor with median survival around 6–12 months, cytoreductive surgery with hyperthermic intraperitoneal chemotherapy (CRS-HIPEC) has been shown to prolong survival in select patients [10]. The oncogenic mechanisms underlying PM remain incompletely understood and are likely multifactorial. Although asbestos exposure is a well-established risk factor for pleural mesothelioma, up to 67 % of patients with PM have no reported history of asbestos exposure, implying that other factors may contribute to pathogenesis [11]. While familial clustering and germline mutations such as *BAP1* support a role for genetic susceptibility, many patients lack a family history or identifiable mutations.

Mesothelial tissue and the genitourinary (GU) tract are embryologically derived from the mesoderm, which gives rise to the coelomic epithelium, visceral peritoneum and the mesodermal ridge (the common origin of the urinary and genital systems) [1], 12]. Notably, the epithelial subtype of mesothelioma is known to closely resemble the histologic morphology of serous papillary carcinomas of the ovary [13]. Numerous case reports describe PM involving the pelvic organs, including complete and partial replacement of the uterus and ovaries, bladder invasion and perforation [8], [14], [15], [16]. Like urogynecologic malignancies, PM growth and progression may be influenced by hormone signaling. Estrogen receptor expression has been linked to survival and one reported case even demonstrated partial response to endocrine therapy [17], 18]. Germline *BAP1* mutations have been found to be associated with both mesothelioma and renal cell carcinoma (RCC) [19].

Although environmental carcinogens, cellular dysregulation, and inherited genetic factors have been implicated in the pathogenesis of both peritoneal mesothelioma and genitourinary malignancies, their co-occurrence remains underexplored. In this study, we present a case series of patients with dual diagnoses and describe post-CRS-HIPEC outcomes between those with and without associated genitourinary cancers.

## Materials and methods

We retrospectively reviewed a prospectively maintained institutional database of patients who underwent CRS-HIPEC between 2011 and 2024. This study was exempt from review by the local Institutional Review Board and informed consent waived. Only cases of histologically confirmed PM were included. Patients with a history of a GU malignancy (including prostate, RCC, testicular, ovarian, endometrial, cervical cancer) were identified based on a comprehensive review of the patient’s electronic medical record. Baseline patient demographics, medical history, and operative data were reviewed in patients with and without associated GU malignancies. Details on type of GU malignancy, treatment received, timeline of diagnosis, asbestos exposure, genetic testing, family history and immunostaining were collected. Descriptive comparisons between groups included peritoneal cancer index (PCI), overall survival, and progression-free survival of PM following CRS-HIPEC. Continuous data were compared using the Wilcoxon rank-sum test for non-parametric data, and categorical data were compared using chi-squared tests. Statistical significance was defined as p<0.05. Data analysis was performed using Stata Statistical Software version 18.0 (Stata Corp., College Station, TX, USA).

## Results

### Case series

Of the 17 patients with PM who underwent CRS-HIPEC, 8 (47.1 %) had another GU malignancy. The median age among patients with both PM and GU malignancies was 53 years (IQR 38–62; range 20–75). There was a slight male predominance (62.5 %) and most patients were White (75 %).

**Figure 1:**
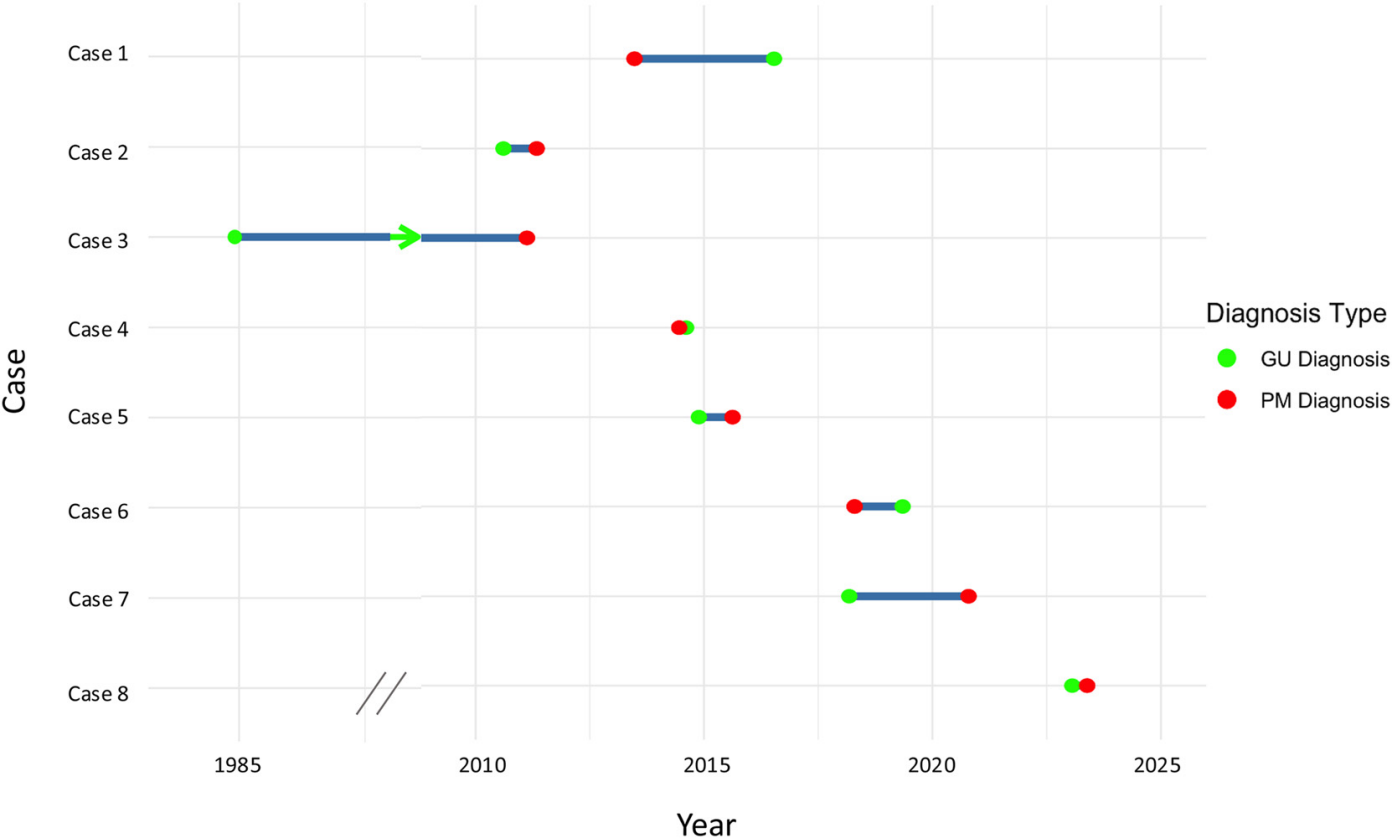
Timeline of peritoneal mesothelioma diagnosis and genitourinary malignancy diagnosis for each case abbreviations: GU, genitourinary; PM, peritoneal mesothelioma.

Details of the 8 cases are outlined in Supplementary Table 1. Associated GU cancers included ovarian neoplasms (n=2; one serous cystadenoma, one mature cystic teratoma of the ovary), cervical carcinoma (n=1), prostate adenocarcinoma (n=3) and RCC (n=2). Most GU malignancies (5/8) were diagnosed prior to PM diagnosis, two were diagnosed after, and one was identified synchronously. The timeline and interval between PM and GU cancer diagnosis is depicted in Figure 1.

The majority (5/8) had epithelioid PM histology, with three cases of well differentiated papillary mesothelioma (WDPM). Clinically, 5 patients presented with abdominal symptoms (including abdominal discomfort, bloating, ascites and/or diarrhea), while 3 PMs were discovered incidentally during unrelated surgeries (an inguinal hernia repair, aortobifemoral bypass and a robotic radical prostatectomy for prostate cancer).

Prior to the diagnosis of PM, only one patient received radiation for treatment of their associated GU malignancy (cervical carcinoma). Three patients had known asbestos exposures (2 with prostate cancer, 1 with RCC). Although 6/8 patients did not have genetic testing available, two patients were found to carry *BAP1* mutations. Four patients had a significant family history of cancer: case 2 (father with lung cancer and uncle with colorectal and kidney cancer), case 3 (mother with early onset gynecologic cancer with metastases to the intestine and sister with lung cancer), case 4 (mother and grandmother with kidney cancer) and case 7 (mother who died of mesothelioma, brother and aunt with kidney cancer).

Immunohistochemical staining was performed in 7 of 8 patients. All were calretinin-positive. *MOC-31* was used in 5 cases (4 negative, one weak focal positivity). Four patients were positive for *D2-40*, three positive for *WT-1* and three were negative for *BER-EP4*. Additional stains included *CK8/18, CK5/6, EMA, CD117, DOG1, p16, CEA, CK7, CK20, PSA, CAM 5.2, CDX2*, and *SATB2*. Full staining results are available in Supplementary Table 2.

### Comparison to patients without GU malignancies undergoing CRS-HIPEC

Demographic characteristics of patients with and without GU malignancies are summarized in Table 1. Patients with associated GU malignancies tended to be younger (52.8 vs. 60.8 years) and more frequently male (62.5 vs. 44.4 %). They also tended to have a lower median Charlson Comorbidity Index (7 vs. 9), and higher rates of smoking (25 vs. 0 %). Otherwise BMI, ASA class, and pre-operative albumin levels appeared similar. Inflammatory markers were generally elevated in patients with GU malignancies relative to those without (C-reactive protein to albumin ratio median 1.5 vs. 0.7, neutrophil to lymphocyte ratio 2.4 vs. 1.2, and monocyte to lymphocyte ratio 0.5 vs. 0.3). There was a higher number of patients in the associated GU malignancy group who had WDPM (3 vs. 0 in the group without). In the group of patients without associated GU malignancy, histology included 6 epithelioid PM, 1 epithelioid (tubulo-papillary subtype), 1 benign multicystic and 1 biphasic PM. Most PM patients without an associated GU malignancy received neoadjuvant chemotherapy (77.8 vs. 25 %, p=0.030) and similar numbers of patients in both groups received adjuvant chemotherapy (33.5 vs. 37.5 %, p=0.86). Among the patients with associated GU malignancies, 0/3 (0 %) of those who received adjuvant chemotherapy died within 3 years, compared to 3/5 patients (60 %) who did not receive adjuvant therapy. Similarly, no patient (0/2, 0 %) who received neoadjuvant chemotherapy died within 3 years, whereas 3/6 (50 %) patients who did not receive neoadjuvant treatment did. Among these 3 patients who experienced 30 day mortality without neoadjuvant or adjuvant therapies, 2/3 (67 %) died in the setting of disease recurrence. For the patients without GU malignancies, regardless of receipt of neoadjuvant or adjuvant chemotherapy, 0/9 patients in this group as a whole experienced mortality within 3 years. Among the 3 patients without GU malignancies who experienced disease progression, one received neoadjuvant chemotherapy and one received both neoadjuvant and adjuvant chemotherapy.

**Table 1:** Demographics and patient characteristics.

	No GU malignancy	Associated GU malignancy
	(n=9, 52.9 %)	(n=8, 47.1 %)
Sex (n, %)		
Male	4 (44.4 %)	5 (62.5 %)
Female	5 (55.6 %)	3 (37.5 %)
Age (years, at time of CRS-HIPEC, median, IQR)	60.8 (51.9, 70.5)	52.8 (38.5, 61.7)
Race (n, %)		
Caucasian	8 (88.9 %)	6 (75.0 %)
African–American	1 (11.1 %)	2 (25.0 %)
BMI (kg/m^2^, median, IQR)	29.4 (23.9, 35.8)	26.0 (25.2, 28.5)
ASA class (median, IQR)	3.0 (3.0, 3.0)	3.0 (3.0, 3.0)
Charlson comorbidity complex (median, IQR)	9.0 (6.0, 9.0)	7.0 (6.0, 8.5)
Smoking history (n, %)		
No	8 (100.0 %)	6 (75.0 %)
Yes	0 (0.0 %)	2 (25.0 %)
Albumin (median, IQR)	4.3 (4.1, 4.4)	4.1 (3.5, 4.4)
CAR (median, IQR)	0.7 (0.4, 3.2)	1.5 (0.3, 3.8)
NLR (median, IQR)	1.2 (0.9, 4.0)	2.4 (2.2, 4.7)
MLR (median, IQR)	0.5 (0.1, 0.6)	0.3 (0.1, 1.0)

Median and 25th, 75th quartiles for continuous variables. GU, genitourinary; CRS-HIPEC, cytoreductive surgery with hyperthermic intraperitoneal chemotherapy; IQR, interquartile range; BMI, body mass index; ASA, American Society of Anesthesiologists; CAR, C-reactive protein to albumin ratio; NLR, neutrophil to lymphocyte ratio; MLR, monocyte to lymphocyte ratio.

Operative details and outcomes of patients with and without associated GU malignancies are summarized in Table 2. At the time of CRS-HIPEC, although there was no statistically significant difference, mean PCI was found to be higher in the patients with associated GU malignancies (19.8 vs. 14.3, p=0.19) and frequency of complete cytoreduction appeared to be slightly lower (62.5 vs. 75 %, p=0.59). Although one-year survival rates were similar across groups, the three-year survival rate was lower among patients with GU malignancies (62.5 vs. 100 %). Notably, all three deaths within 3 years in the cohort occurred in the GU malignancy group, with causes including septic shock due to bowel perforation in the setting of recurrent mesothelioma, multisystem organ failure from necrotizing soft tissue infection 10 days post- CRS-HIPEC, and recurrent PM with bowel obstruction. Disease progression was observed in 4 patients in the GU malignancy group: at 14, 18, 25 and 26 months post-HIPEC and in 3 patients in the non-GU malignancy group at 2, 10 and 21 months.

**Table 2:** Operative details and outcomes.

	No GU malignancy	Associated GU malignancy	p-Value
PCI (average ± SD)	14.25 ± 4.5	19.75 ± 10.5	0.194
Cytoreduction score (n, %)			0.590
0	6 (75.0 %)	5 (62.5 %)	
1	2 (25.0 %)	3 (37.5 %)	
1-Year overall survival (n, %)	9 (100 %)	6 (75 %)	0.110
3-Year overall survival (n, %)	9 (100 %)	5 (62.5 %)	**0.043**
Progression at 1 year (n, %)	3 (33.3 %)	2 (25.0 %)	0.707
Progression at 3 years (n, %)	3 (33.3 %)	4 (50.0 %)	0.486

Bold represents statistical significance. PCI, peritoneal carcinomatosis index; SD, standard deviation.

### Literature review

We conducted a comprehensive review of the literature to assess the prevalence of other reported cases of PM associated with GU malignancies, summarized in Supplementary Table 3. In one study of 500 patients with asbestos-related malignant mesotheliomas, only 10 (1.8 %) had additional primary malignancies in general. The majority of these mesotheliomas were pleural, with just one peritoneal case [20]. Among these, six of the secondary tumors were primary bronchogenic carcinomas and the remaining were colonic, pancreatic and breast adenocarcinomas. In a population based-study of 895 PM and 3,672 pleural mesotheliomas, 88 (9.8 %) PM patients had a prior primary cancer and 28 (3.1 %) developed a subsequent malignancy. Among the prior cancers, GU tumors accounted for 26.1 % (including cervical, endometrial and prostate), while GU malignancies made up 17.8 % of second primaries (prostate and endometrial). A bidirectional association was observed between pleural mesothelioma and kidney cancer, implicating potential genetic mechanisms such as *BAP1* mutations [21]. In another study of 64 PM patients, 22 % had at least one additional malignancy, including ovarian cancer (14 %) and RCC (7 %) [22]. Malpica et al. reported that 31.1 % of women with PM had a history of other cancers, with 73.6 % of those being GU in origin (ovarian, endometrial, cervical and kidney) [23]. Only 9 % had documented asbestos exposure, and genetic testing was limited to 5 patients (1 *BAP1* mutation, 1 type 2 neurofibromatosis, 1 Lynch syndrome, 1 McCune-Albright and one negative) [23].

Several case reports describe additional cases of associated PM and GU cancers, including four cases with endometrial cancer (three WDPM, one epithelioid histology with Lynch syndrome), two with serous ovarian tumors, and one with cervical carcinoma [24], [25], [26], [27], [28], [29]. In a SEER database study of 570,883 patients with a history of prostate cancer, Farioli et al. identified 471 mesothelioma cases, however only 21 of these were peritoneal mesotheliomas [30]. Other case reports document PM occurring alongside testicular seminoma (n=2), renal cell carcinoma (n=3), and urothelial carcinoma of the bladder (n=3) [31], [32], [33], [34], [35], [36], [37], [38]. One series of patients with synchronous gastrointestinal stromal tumor (GIST) and PM also included cases with concurrent RCC, prostate and ovarian serous cystadenoma [39].

## Discussion

In this series of 17 patients with PM undergoing CRS-HIPEC, nearly half (47 %) had a co-occurrence of GU malignancy, with one synchronous case and 7 metachronous cases. The most common associated malignancy was prostate adenocarcinoma, followed by RCC and ovarian tumors. Patients with GU malignancies tended to have high PCI scores, suggesting the possibility of a greater disease burden. While progression rates were similar between groups, the 3-year overall survival rate was lower in patients with GU malignancies. Given that the incidence of GU cancers in the US is estimated to be 597,160 cases annually (around 0.18 % of the US population each year), our findings represent a potentially higher than expected number of GU malignancies diagnosed in PM patients undergoing CRS-HIPEC [40]. These exploratory findings therefore raise the possibility of a non-random association between these two cancers.

Peritoneal mesothelioma remains rare in population-based studies, with 1998 new cases identified in the Surveillance, Epidemiology and End Results (SEER) database from 1975 to 2016, and age-adjusted incidence remaining stable over time (1.02 cases/million) [41], 42]. Consistent with our cohort, these studies reported PM to be slightly more common in men, Caucasians, and patients older than 50 years. Epithelioid histology was the most frequent subtype and increased by 240 % over 18 years, although histologic data was largely unavailable. Approximately 50.6 % of patients received chemotherapy, and the proportion undergoing cancer-directed surgery increased from 27 to 43 % over time. Receipt of cancer-directed surgery and more recent diagnosis were associated with improved survival [41], 42]. These studies do not describe the use of CRS-HIPEC or concurrence of other malignancies, areas explored in our series.

Multiple etiologic factors may underlie a possible association between PM and GU malignancies, including environmental exposures, genetic predispositions, hormonal influences and inflammatory factors. Asbestos exposure has long been an established risk factor for pleural mesothelioma, with up to 80 % of cases linked to asbestos and approximately 5 % of asbestos-exposed workers developing mesothelioma [43]. However, this link is less pronounced for PM, particularly among women [44]. An estimated 20 % of mesothelioma patients are without known prior asbestos exposures (and among this group PM is more common) suggesting that other factors may contribute to pathogenesis [11], 31]. In our cohort 3 of 8 patients (37.5 %) reported a known history of asbestos exposure. Within our literature review, only three patients with PM had a known history of asbestos exposure and associated GU malignancy (albeit exposures existed in other studies but were not definitively allocated to specific patients with PM and GU malignancies). While some studies have linked asbestos to the development of GU cancers as well, evidence remains inconclusive [32], 45]. For example, a large meta-analysis by Franco et al. found no increased risk of bladder cancer among individuals with occupational asbestos exposure [45].

Radiation exposure is another recognized risk factor for the development of malignancy. Cases of PM have been reported following pelvic radiation for other malignancies [3], 16], 20], 29]. In one SEER study, external beam radiation for prostate cancer was associated with a modest increased risk of developing PM (65.0/100,000 over 15 years) [30]. However, there remained 281/471 (59.6 %) prostate cancer patients in this study who were not treated with radiation yet developed mesothelioma. In our cohort, only one patient received radiation (for cervical cancer) prior to the development of PM.

Inflammation and trauma have been cited as additional suspected risk factors for the development of mesothelioma. Testicular mesothelioma has been linked to prior hernia repairs, long term hydrocele, epididymitis and orchitis [3], 4]. Endometriosis has been associated with at least 23 cases of PM in the literature [46], 47]. Chronic inflammatory states may promote mesothelioma development via persistent cytokine signaling – particularly IL-6, which is also implicated in the tumor microenvironment of prostate, renal and gynecologic malignancies [48]. Although specific inflammatory processes were not assessed in our cohort, patients with associated GU cancers tended to have higher inflammatory markers (CAR, NLR, MLR), suggesting a potential tumor-promoting inflammatory milieu.

Hormonal signaling may also play a role in the pathogenesis of PM. Women with PM tend to have better survival outcomes than men, possibly due to estrogen receptor signaling [49], 50]. One reported case of pleural epithelioid mesothelioma with concurrent breast cancer even demonstrated a sustained response to endocrine therapy [18].

Genetic predisposition is likely another important factor. *BAP1* is the best known gene linked to hereditary mesothelioma, and other genes (i.e. *NF2*, *TP53*, *CDKN2A*, *SETDB1*, and *SETD2*) are increasingly being identified [51]. In our series, two patients with GU malignancies had confirmed germline *BAP1* mutations, and half had strong family histories of cancer. Together with prior reports, these findings support a possible underlying genetic susceptibility to multiple malignancies in this patient population.

The prevalence of GU malignancies in our cohort of patients with PM undergoing CRS-HIPEC suggests a potential non-random association between these cancer types. A link between the two could stem from shared embryologic origins, environmental exposure, inflammatory tumor microenvironments or unrecognized genetic drivers. CRS-HIPEC is associated with a significant survival benefit in PM, increasing median survival to 5 years compared to 1 year without [52]. In our cohort, 62.5 % of patients with GU malignancies were alive at 3 years, supporting the continued use of CRS-HIPEC in this population. However, differences in inflammatory markers, PCI and survival rates when compared to PM cases without associated GU cancers suggests that this group may represent a more locally aggressive disease subset. This hypothesis merits further study of the biological behavior of PM, comparative aggressiveness, and prognostication following CRS-HIPEC. The role of systemic chemotherapy is less well defined for patients with PM [52]. Although our cohort is small, all of the patients with associated GU malignancies who died within 3 years of CRS-HIPEC did not receive neoadjuvant or adjuvant therapy. Further investigation into the benefits of systemic therapies for this group may be warranted. Finally, these findings highlight the need for a high index of suspicion for genetic mutations and low threshold for genetic testing in these patients, particularly those with a personal or family history of cancer. Further, they underscore the importance of continued research into additional genetic alterations that may contribute to the development of PM and other malignancies.

This study has several limitations, including its small sample size and retrospective, exploratory design which does not allow for establishment of a definitive epidemiological link. These findings are rather hypothesis generating, recognizing that a true epidemiologic study design may be challenging for this rare disease. As we focused on patients undergoing CRS-HIPEC, our cohort primarily included those with epithelioid PM (and also included well-differentiated papillary mesothelioma) – both of which are less aggressive histologic subtypes – and patients healthy enough to undergo major surgery. Therefore, findings may not generalize to the broader PM population, including those with biphasic or sarcomatoid histology. Additionally, the GU malignancies represented were heterogenous in type and clinical behavior. Both synchronous and metachronous cancers were included as we wished to broadly understand the presence of any GU malignancies before or after PM diagnosis. As such, causal relationships between PM and GU cancers cannot be inferred. The comparative analysis of patients with and without GU malignancies is underpowered due to small sample size and therefore reported statistics should be interpreted descriptively rather than analytically. Finally, the small sample size precluded multivariable survival analysis, limiting our ability to adjust for potential confounders that may influence observed outcome differences.

In summary, we present a case series highlighting a previously undocumented high rate of GU malignancies in patients with PM undergoing CRS-HIPEC. While not epidemiologically definitive, these findings generate a seminal hypothesis regarding a connection between these two malignancies and highlight the need for further research into shared risk factors and personalized therapeutic approaches. As PM remains a rare and complex disease, findings such as these create a foundation for further study to help to refine patient selection, guide diagnostic workup and optimize management strategies.

## Supplementary Material

Supplementary Material

Supplementary Material

Supplementary Material
