# Supplementary material for: Co-occurrence of peritoneal mesothelioma and genitourinary cancers: a case series with comparative outcomes
Source: Pleura Peritoneum. 2025 Nov 3;10(4):177–84. doi: 10.1515/pp-2025-0020 (PMC12707186; doi:10.1515/pp-2025-0020)
Supplement: Supplementary file 1 — Supplementary Material [file j_pp-2025-0020_suppl_001.docx]

**Supplemental Table 1:** Cases of Peritoneal Mesothelioma with Associated GU Malignancy

| Case | Age  (at CRS-HIPEC) | Peritoneal histology | Urothelial malignancy | Treatment of GU Malignancy | Asbestos Exposure | Genetic Testing |
| --- | --- | --- | --- | --- | --- | --- |
| 1 | 34 | WDPM | Serous cystadenoma left ovary | Salpingo-ophorectomy | None | None performed |
| 2 | 42 | Epithelioid | Papillary RCC | Partial nephrectomy | None | None performed |
| 3 | 64 | Epithelioid | Cervical carcinoma | TAH-BSO + adjuvant radiation | None | None performed |
| 4 | 19 | Epithelial (papillary/ tubulo-papillary pattern | Mature cystic teratoma ovary | Salpingo-ophorectomy | None | *BAP1* VUS |
| 5 | 49 | WDPM | Well differentiated prostatic adenocarcinoma | Conservative | Yes | None performed |
| 6 | 55 | WDPM | Metastatic prostatic adenocarcinoma | Chemotherapy | None | None performed |
| 7 | 58 | Epithelioid | Clear cell renal carcinoma | Partial nephrectomy & chemotherapy | Yes | *BAP1, ARID1A, ASXL1* |
| 8 | 74 | Epithelioid | Prostatic adenocarcinoma | Plan for RALP, aborted due to peritoneal implants | Yes | None performed |

Abbreviations: GU, genitourinary; CRS-HIPEC, cytoreductive surgery with hyperthermic intraperitoneal chemotherapy; WDPM, Well differentiated papillary mesothelioma; RCC, renal cell carcinoma; TAH-BSO, total abdominal hysterectomy and bilateral salpingo-oophorectomy; RALP, robotic-assisted laparoscopic prostatectomy.
