# Supplementary material for: Co-occurrence of peritoneal mesothelioma and genitourinary cancers: a case series with comparative outcomes
Source: Pleura Peritoneum. 2025 Nov 3;10(4):177–84. doi: 10.1515/pp-2025-0020 (PMC12707186; doi:10.1515/pp-2025-0020)
Supplement: Supplementary file 2 — Supplementary Material [file j_pp-2025-0020_suppl_002.docx]

**Supplemental Table 2:** Immunohistochemical Stain Results by Case

| Case | *Calretinin* | *D2-40* | *WT1* | *MOC-31* | *CK5/6* | *CK7* | *AE1/ AE3* | *CAM5.2* | *CK20* | *BerEP4* | *P16* | *PSA* | *RCC Ag* | *BAP1* | *CDX2* | *EMA* | *DOG1* | *CEA* | *SATB2* | *CK 8/18* |
| --- | --- | --- | --- | --- | --- | --- | --- | --- | --- | --- | --- | --- | --- | --- | --- | --- | --- | --- | --- | --- |
| 1 | + | + |  | – | + |  |  |  |  |  | <1% |  |  |  |  | – | – |  |  | + |
| 2 | + | + |  | (+) | + |  |  |  |  | – |  |  | + |  |  |  |  | - |  |  |
| 3 | + |  |  |  |  | + |  |  | – |  |  |  |  |  |  |  |  |  |  |  |
| 4 | + |  |  | – |  |  |  |  |  |  | + |  |  |  |  |  |  |  |  |  |
| 5 | + |  | + |  |  |  | + |  |  |  |  | – |  |  |  |  |  |  |  |  |
| 6 |  |  |  |  |  |  |  |  |  |  |  |  |  |  |  |  |  |  |  |  |
| 7 |  | + | + | – |  | + |  | + | – | – |  |  |  |  | – |  |  |  | – |  |
| 8 | + | + | + | – |  |  | + |  |  | – |  |  |  | Retained |  |  |  |  |  |  |

(+) indicates weak focal positivity
