# Supplementary material for: Co-occurrence of peritoneal mesothelioma and genitourinary cancers: a case series with comparative outcomes
Source: Pleura Peritoneum. 2025 Nov 3;10(4):177–84. doi: 10.1515/pp-2025-0020 (PMC12707186; doi:10.1515/pp-2025-0020)
Supplement: Supplementary file 3 — Supplementary Material [file j_pp-2025-0020_suppl_003.docx]

**Supplemental Table 3:** Summary of Reported Cases of Peritoneal Mesothelioma and Genitourinary Malignancies

| Study | Study Design / N | GU Malignancies Reported | Key Findings | Asbestos Exposure |
| --- | --- | --- | --- | --- |
| Chen et al.[21] | Population-based SEER study (895 peritoneal & 3672 pleural mesothelioma) | 23 1st GU (5 cervical, 9 endometrial, 9 prostate), 5 2nd GU (3 prostate, 2 endometrium) | 9.8% with prior primary cancer before PM; bidirectional association between pleural mesotheliomas and kidney cancer | Unknown |
| Pandav et al.[22] | Retrospective study of 64 PM cases | 2 ovarian (14%), 1 RCC (7%) | 14 patients (22%) had ≥1 additional malignancy | 3/14 patients |
| Malpica et al.[23] | Retrospective study of 122 female PM patients | 28 GU (70% of prior malignancies): 17 ovarian, 5 endometrial, 3 cervical, 2 vaginal, 1 kidney | 31.1% had prior malignancies, mostly GU | 11/122 patients |
| Lu et al. [24] | Case report | Endometrial adenocarcinoma | Patient with Lynch Syndrome developed epithelioid PM 10 months after uterine endometroid adenocarcinoma | Unknown |
| Lewis et al. [25] | Case report | Endometrial adenocarcinoma | Synchronous with PM (WDPM) | Unknown |
| Chen et al. [26] | Case report | Endometrial adenocarcinoma | Synchronous with PM (WDPM) | None |
| McGinnis et al.[27] | Case report | Endometrial serous carcinoma and ovarian high-grade serous carcinoma | Synchronous with PM (WDPM) | Unknown |
| Losi et al.[28] | Case report | Serous borderline ovarian tumor | Epithelioid PM 1 year after serous borderline ovarian tumor | None |
| Yano et al.[29] | Case report | Cervical squamous cell carcinoma | Epithelioid MPM 1 year after cervical cancer | None |
| Farioli et al.[30] | SEER-based study (570,883 prostate cancer pts) | 21 PM + prostate cancer | IRR of PM was 3.87 (95% CI 0.65-23.1) for latency periods of 10 years or more after EBRT | Unknown |
| Manzini et al.[31] | Retrospective multi-institutional study (81 PM patients) | 1 testicular seminoma, 1 bladder and 1 prostate | 8.6% (7/81) PM patients with a diagnosis of another malignancy during lifetime | Asbestosis in 15 cases |
| Candura et al.[32] | Case report | RCC | Biphasic PM 1 year after diagnosis of RCC treated with surgery | Asbestos exposure |
| Kawakita et al.[33] | Case report | RCC | Synchronous with PM | None |
| Murinello et al.[34] | Case report | RCC | Synchronous with PM (epithelial type) | Asbestos exposure |
| Basatac et al. [35] | Case report | Bladder cancer | Synchronous with PM | None |
| Brahim et al.[36] | Case report | Bladder cancer | Synchronous with PM (epithelioid) | Asbestos exposure |
| Tassile et al. [37] | Case report | Testicular seminoma | Presented with PM (mix of epithelioid and fusiform cells) & colon cancer 29 years after radiation for testicular cancer | None |
| Obrist et al. [38] | Case report | Small cell prostatic cancer | Synchronous with PM (epithelial) | Unknown |
| Courelli et al.[39] | Case series (8 patients with GIST & PM) | 1 RCC, 1 prostate, 1 ovarian serous cystadenoma | Synchronous with PM | Unknown |

Abbreviations: GU, genitourinary; PM, peritoneal mesothelioma; SEER, surveillance epidemiology and end results; RCC, renal cell carcinoma; WDPM, well-differentiated peritoneal mesothelioma; GIST, gastrointestinal stromal tumor.
